# Highly Biocompatible Nanoparticles of Au@Fluorescent Polymers as Novel Contrast Agent for In Vivo Bimodality NIR Fluorescence/CT Imaging

**DOI:** 10.1155/2019/8085039

**Published:** 2019-06-10

**Authors:** Mengshu Zhang, Jinlei Liu, Guannan Wang

**Affiliations:** ^1^First Affiliated Hospital of Jinzhou Medical University, Jinzhou 121001, China; ^2^The Key Laboratory for Medical Tissue Engineering, College of Medical Engineering, Jining Medical University, Jining 272067, China

## Abstract

In this work, one kind of biocompatible and all-in-one dual-modal nanoprobe, based on Au nanoparticles and NIR emissive semiconducting fluorescence polymers, was developed by the one-step solvent-mediated self-assembly method for *in vivo* X-ray computed tomography (CT) and fluorescence bioimaging for the first time. After preparation, a series of comprehensive evaluations were performed, and the nanoprobe exhibited smart size and modification, good compatibility, inducement of autophagy, long blood circulation, unconspicuous *in vivo* toxicity, and excellent fluorescence/CT imaging effects. Overall, the studies in this work assuredly indicate that the synthesized Au@FP nanoparticles as a noninvasive contrast agent is suitable for *in vivo* fluorescence/X-ray CT bimodality biomedical imaging and diagnosis.

## 1. Introduction

*In vivo* imaging, which enables us to peer deeply within living subjects, is producing tremendous opportunities both for clinical diagnostics and as a research tool. In recent years, nanoprobes have exhibited obvious advances in biomedical applications to present comprehensive information for single or combined modalities imaging diagnosis [1–7].

Among them, CT has long been a common technique in clinical imaging in terms of availability, efficiency, and cost, and it also could give high spatial and temporal resolution 3D structure details of tissues with differential X-ray absorption features. However, CT imaging is always used traditionally for bone imaging because CT easily distinguishes between electron-dense structures (high-Z materials, e.g., bone) and relatively electron-poor entities (e.g., soft tissues) [8, 9]. Meanwhile, the clinically used CT contrast agents are small organic iodinated molecules, which are problematic because they are subject to short circulation and imaging time and even potential renal damage by their rapid kidney's clearance [1, 10, 11]. The emergence of nanomaterials provides a possible way to solve the above-mentioned problem [12–15]. Notably, gold nanoparticles are promising from the viewpoint of its large atomic number, X-ray absorption coefficient, facile modification, long circulation time, and good biocompatibility. These advanced properties allow both improved diagnostic accuracy and sensitivity over multiple days without multiple contrast administrations.

In spite of the advantages of CT, it still has its inhabit shortage: the low sensitivity to the soft tissues and unable of cellular or molecular imaging [1]. Fluorescence-based imaging (FI) techniques offer rapid and sensitivity imaging compared to CT imaging. And for *in vitro* applications, most standard fluorescence microscopes offer fast image acquisition on the millisecond timescale allowing many cellular processes to be imaged in real-time. The appropriate fluorescent nanoprobes could be used to provide real-time visualization of the molecular edge between tumor and adjacent normal tissue and consequent identification of the adequate tumor margin before surgery [16–18]. A great challenge for *in vivo* fluorescence imaging is the autofluorescence and strong attenuation in visible light (400 nm∼650 nm) by biological matter, rendering deep tissue imaging all but impossible in this spectral region. Therefore, using mostly established fluorescent nanoprobes such as inorganic quantum dots (QDs) [19], organic dyes [20], and semiconducting conjugated fluorescent polymers [21, 22] with higher transparency in the near-infrared biological windows region (NIR, from 650 nm to 950 nm) has been developed extensively for deep tissue imaging applications. While compared with inherent disadvantages of QDs and organic dyes, such as photobleaching and potential toxicity [23–25], conjugated fluorescent polymers are highly demanded owing to their high brightness, low toxicity, stable fluorescent imaging, deeper tissue penetration, distinguishablility from autofluorescence [18, 21, 26]. Therefore, uniting CT and fluorescence for bimodality imaging is an ideal technique that overcomes the shortcomings of each method alone and provides high-resolutional/sensitive imaging of tissue and cells. Although the CT/fluorescence bimodality imaging can provide more accurate information for diagnosis, it is still a big challenge to develop facile methods to synthesize an all-in-one smart multifunctional nanoprobe for satisfied and effective CT/fluorescence bimodality imaging.

**Scheme 1:**
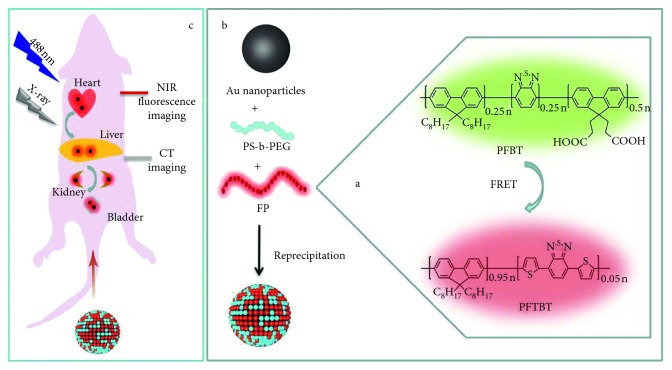
Illustration of the fabrication procedure of Au@FP nanoparticles and their application for dual-modal imaging.

Herein, we developed such kind of all-in-one multifunctional nanoparticles (Au@FP nanoparticles) by one-step solvent-mediated self-assembly method and then explored for its potentials as a contrast agent for both CT/fluorescence imaging. The components of hydrophobic Au nanoparticles core, NIR emitting conjugated fluorescence polymers (PFBT and PFTBT), and amphiphilic PEGylated polymers are integrated into a single particle by rapid one-step synthesis (Scheme 1). The Förster resonance energy transfer (FRET) strategy between fluorescence polymers was applied to enhance NIR fluorescence. Also, PEGylated amphiphilic polymer was used in synthesis to prominently improve the water solubility, circulation time, and biocompatible [21, 26]. After preparation, a series of comprehensive evaluations are performed to characterize their size, morphology, cytotoxicity, autophagy, and CT/fluorescence imaging effects *in vitro* and *in vivo*. Furthermore, we investigated the role of serum biochemistry assay for evaluating nanotoxicity; the results do not reveal any dysfunction in terms of renal function. Overall, based on the above results, it is obvious that the designed Au@FP nanoparticles possess great biocompatibility and promising application for CT/fluorescence bimodality imaging.

## 2. Materials and Methods

### 2.1. Materials and Instruments

All chemicals were purchased from Sigma-Aldrich unless otherwise noted. The morphology and size were characterized by transmission electron microscopy (TEM, FEI TECNAI G20). Elements analysis was conducted on an Agilent 7500a Series. A Malvern Zetasiser NANO ZS was employed to measure dynamic light scattering (DLS). Also, the binding energy scales of the spectra for XPS measurements were using a model Thermo ESCALAB 250XI.

### 2.2. Synthesis of Au Nanoparticle@Fluorescence Polymers (Au@FP) Nanoparticles

Hydrophobic Au nanospheres (Au) with 5 nm diameter were synthesized through chemical reduction of HAuCl_4_ according to a previous report [27]. Meanwhile, *π*-conjugated fluorescent polymer (FP) of PFBT and PFTBT with red emission at 660 nm wavelength was prepared according to previous work [28], and their structures and mechanism of FRET strategy are shown in Scheme 1(a). AuNPs, PFBT, PFTBT, and PEGlayted amphiphilic block copolymer (poly(styrene)-block-poly(ethylene glycol), PS-b-PEG) stock solution in tetrahydrofuran (THF) at 5 mg/mL were used for further experiments.

In this work, the hydrophobic AuNPs was first used in the solvent-mediated self-assembly method. As the typical experiments [18, 21], hydrophobic AuNPs, fluorescent polymers, and PS-b-PEG were assembled through the solvent-mediated self-assembly method to synthesize Au@FP nanoparticles. Firstly, 200 *μ*L of AuNPs, 200 *μ*L of PFBT, 100 *μ*L of PFTBT, and 100 *μ*L of PS-b-PEG solution were mixed together. Then, the mixed solution was rapidly injected into 5.4 mL of water under vigorous sonication. The solution gradually turned to a light milky suspension, indicating the formation of Au@FP nanoparticles. Then, nitrogen was used to remove the THF solvent at room temperature. After filtered by 0.2 *μ*m microporous filtration, the stock solution of Au@FP nanoparticles was prepared.

### 2.3. Cell Cytotoxicity Assessment

To quantitatively evaluate the cytotoxicity of the Au@FP nanoparticles, in this study, the astrocytes and human umbilical vein endothelial cells (HUVECs) were used, and the methods used were according to our previous work [18]. Briefly, the cells were seeded at a density of 10^4^ cells per well, and then the cells were incubated with the desired amounts of Au@FP nanoparticles ranging from 10 to 500 *μ*g/mL. After treatment for 24 h, cell cytotoxicity was assessed by standard MTT assay with triplicate times.

To evaluate the ROS activity induced by the Au@FP nanoparticles, HUVECs were seeded into 24-well plates; after being cultured overnight for cell attachment, the cells were treated with H_2_O_2_ (500 *μ*M), Au@FP nanoparticles (200 *μ*g/mL), and PBS, respectively. After one day of culture, the cells were incubated with dichlorofluorescin diacetate (DCFH-DA) in a culture medium for 20 min and were washed by ice cold serum-free cell culture medium three times, and the fluorescence intensity emission at 525 nm was recorded by using a plate reader.

Similar to our previous study [18], for the western-blot test of autophagy, total cellular protein lysates were first collected and separated by SDS-polyacrylamide gel electrophoresis. Then, after transferring onto membranes, the target protein bands were visualized and imaged.

### 2.4. *In Vitro* Cellular Imaging

Briefly, the HUVECs were cultured in chambers (NET); then 200 *μ*L of 200 *μ*g/mL Au@FP nanoparticles were added. After incubation for two hours, free nanoparticles were washed away by a large amount of PBS. Next, 5 *μ*g/mL of 4′, 6-diamidino-2-phenylindole (DAPI) was employed to stain the nucleus for 20 min, and tubulin structure was stained by anti-*α*-tubulin antibody and Alexa Fluor® 488-labeled secondary antibody. Finally, after fixed by 4% paraformaldehyde, the CLSM was employed to record the morphology of cells and track the Au@FP nanoparticles location in the cells.

### 2.5. Animals and Treatment

All animal experiments including relevant details were approved by the Regional Ethics Committee of Jinzhou Medical University, China (permit number: LMU-2013-368). Also, in this work, male Sprague-Dawley rats (180–200 g) were used for *in vivo* study.

### 2.6. CT Image and Biodistribution

For *in vitro* CT imaging, the Au@FP nanoparticles in PBS buffer solution were dispersed at concentrations from 5 *μ*g/mL to 500 *μ*g/mL. For *in vivo* CT imaging, the SD rats (control and test rats) were selected. At first, we anesthetised the SD rat by intraperitoneal injection of chloral hydrate solution (10 wt.%), and then 200 *μ*L of Au@FP nanoparticles solution (200 *μ*g/mL) was injected via the tail vein. CT images were collected using an LN U.A NO.1HOSP Philips iCT 128 slice scanner. Imaging parameters were as follows: thickness, 2.5 mm; pitch, 0.9; 120 kVp, 500 mA; and table speed, 190 mm/s.

*In vivo* NIR fluorescent images of the supine position and the time-dependent biodistribution of Au@FP nanoparticles in mice before injection and at 3 h and 6 h after injection were taken using a whole body optical imaging system with an excitation filter (480–40*x*) and an emission filter (615–665 M). Autofluorescence was removed by using spectral unmixing software.

### 2.7. Histological Study

For hematoxylin and eosin (H&E) study, the test rats were sacrificed, and organs (heart, liver, spleen, lung, kidney, and stomach) were collected. After dehydrated and paraffinic inclusion, the organs were sectioned and stained with H&E; at last, the pictures of histological sections were taken under an Olympus digital camera.

### 2.8. Serum Biochemistry Assay

For the SD rats in the control and test groups, 0.8 mL blood of each rat was collected for serum biochemistry assay and renal function test.

### 2.9. Statistics

The statistical analysis process was implemented on the one-way ANOVA statistical analysis. Average values are presented as mean ± standard deviation (SD), and a *P* value < 0.05 was considered statistically significant.

## 3. Results and Discussion

### 3.1. Synthesis and Characterization of Au@FP Nanoparticles

These Au nanospheres (Au) with 5 nm diameter were synthesized through chemical reduction of HAuCl_4_ according to a previous report [27], and *π*-conjugated fluorescent polymer (FP) of PFBT and PFTBT with NIR emission at 660 nm wavelength was prepared according to previous work [28]. The synthetic process for mutifunctional probes (Au@FP nanoparticles) by solvent-mediated self-assembly method is illustrated in Scheme 1. At first, hydrophobic octanethiol functional Au nanoparticles and fluorescent polymers were mixed with amphiphilic block polymer PS-b-PEG in tetrahydrofuran (THF), which is a good amphiphilic solvent. When they were rapidly added into water under ultrasonication, hydrophobic components will aggregate together due to the hydrophobic-hydrophobic interaction to form into a small “vesicle” coated with a hydrophilic PEG shell. It is formatted by the amphiphilic PS-b-PEG, which allows for sequestration of vesicles inside the hydrophobic core and the hydrophilic outer surface. The outside PEG structures promise their hydrophilic property, long-term circulation time, and low cytotoxicity [29, 30]. At last, elements analysis study reveals that weight percentages of Au encapsulated are 27.5%.

**Figure 1:**
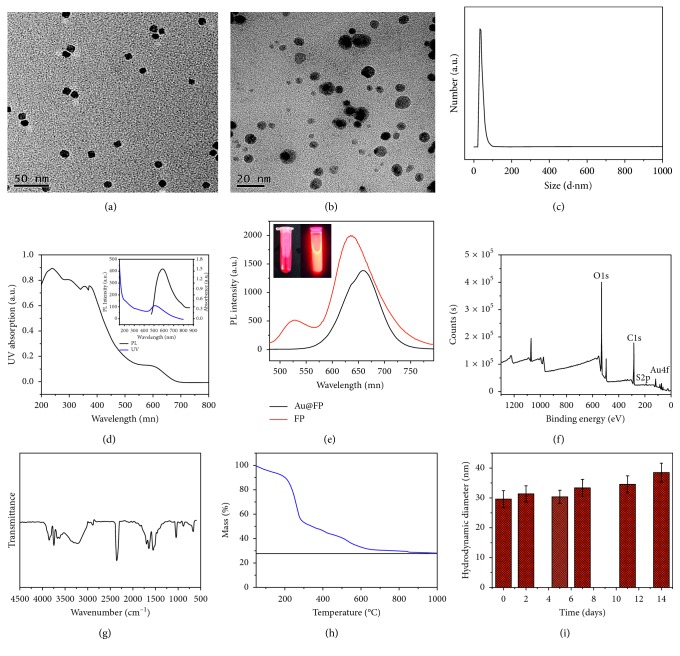
Characterization of the nanoparticles: transmission electron micrographs of Au (a) and Au@FP nanoparticles (b); DLS of Au@FP nanoparticles (c); ultra-vis absorption spectrum of Au@FP nanoparticles, the inset is the fluorescence and ultra-vis absorption spectrum of Au nanoparticles (d); fluorescence spectrum of Au@FP nanoparticles and FP, the inset is digital photo of Au@FP nanoparticles under ultraviolet light (366 nm) and daylight (e); XPS spectra of Au@FP nanoparticles (f); FTIR spectra of Au@FP nanoparticles (g); TGA curve of Au@FP nanoparticles (h); the stability of Au@FP nanoparticles in water during two weeks (i). Values are mean SD; *N* = 3.

For medical imaging, the size of nanoparticles within a certain range is essential. In general, previous works have claimed that particles with sizes ranging from 10 nm to 100 nm are optimal for passive targeting of most types of solid tumors by capitalizing on the enhanced permeability and retention (EPR) effect [10, 18, 31], and size reduction could be helpful to increase the passive targeting effects. The transmission electron microscopy (TEM) images of Au and Au@FP nanoparticles were shown in the Figures 1(a) and 1(b), respectively. The prepared Au nanoparticles showed the ideal sizes around 5 nm with high uniformity (polydispersity index: 0.110). And after self-assembly with fluorescent and block polymers, the final Au@FP nanoparticles exhibited sizes around 20 nm (Figure 1(b)). The average hydrodynamic diameter of Au@FP nanoparticles in water estimated by dynamic light scattering (DLS) was approximate 30 nm with zeta potential of −20.4 ± 0.283 mV (Figure 1(c)), which affirms their small sizes in aqueous solution without any aggregation [32]. As crucial factors for fluorescent imaging, the photoluminescence (PL) and UV-vis absorption spectra of Au@FP nanoparticles are shown in Figure 1(d), and the inset is the PL and UV-vis absorption spectra of Au nanoparticles. As mentioned above, FRET strategy was employed for the NIR emitting polymer (PFTBT) by using green emitting polymer (PFBT) as the energy donor to reduce the self-quenching [21, 28]. From the UV-vis absorption spectra, we can see that there is very strong absorption at around 440 to 450 nm derived from the PFBT. Also, we can see there is mild absorption at around 500 to 600 nm, which should attribute to the abroad absorption of Au nanoparticles. By an efficient energy transfer between the fluorescent polymers, high-intensity emission at around 660 nm was observed, which promises that Au@FP nanoparticles hold great potential for *in vivo* fluorescent imaging. Although fluorescent intensity of Au@FP nanoparticles showed little weakness compared with pure fluorescnece polymers (Figure 1(e)), and the fluorescent peak at 520 nm disappeared, owing to the broad and strong absorption of Au nanoparticles. The inset in Figure 1(e) shows the digital photo of the aqueous dispersion of Au@FP nanoparticles, and it could emit bright red fluorescence, and the solution was the distinguishable deep red color under white light. The composition of the Au@FP nanoparticles was further tested by XPS spectra and FTIR and is shown in the Figures 1(f) and 1(g). The Au 4f high-resolution XPS pattern reveals that the binding energy values of Au4f_7/2_ and Au4f_5/2_ are 83.7 and 87.4 eV, respectively, consistent with the previous reports about Au nanoparticles [33, 34]. The Au4f, C1s, O1s, S2p, and N1s derived from Au nanoparticles and fluorescence polymers confirm the formation of Au@FP nanoparticles. The FTIR spectrum of the Au@FP nanoparticles is shown in Figure 1(g), the characteristic bands at around 1538 cm^−1^ correspond to the coordinated carboxylic acid groups, and peaks at 2300 cm^−1^ was typical for a nitrile stretch (-C=N) derived from the fluorescent polymer, demonstrating the conjugation of Au nanoparticles with polymers. The thermal stability of the Au@FP nanoparticles was also evaluated by TGA in helium atmosphere shown in Figure 1(h), and the TG curve showed good thermal stability with an initial decomposition temperature just below 240°C. The residue weight percentage of Au@FP nanoparticles at 1000°C was about 28% of pure Au, which was consistent with the result of ICP-MS. At last, the stability of the Au@FP nanoparticles was investigated by monitoring the hydrodynamic sizes at room temperature (Figure 1(i)). After 14 days of storage, there was no distinct difference in the size of the Au@FP nanoparticles; they still retained good dispersity without any obvious aggregation.

### 3.2. *In Vitro* CT Imaging and Fluorescence Imaging

**Figure 2:**
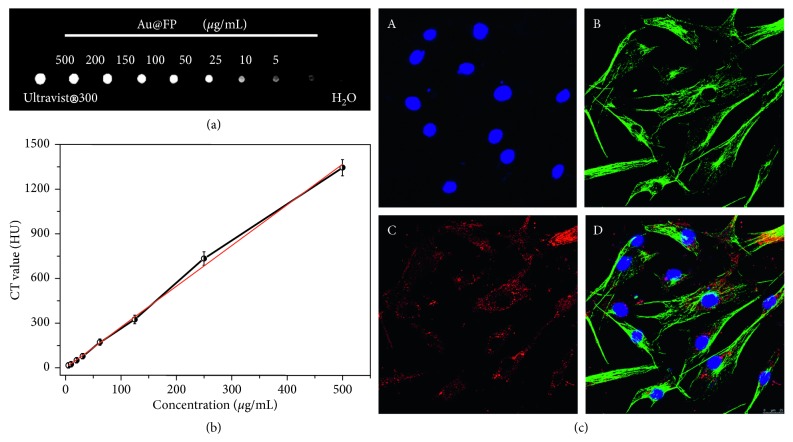
Computed tomography (CT) images of Au@FP nanoparticles and clinical CT contrast agent (Ultravist® 300) of increasing concentration (*μ*g/mL) (a); standard curve of CT values (hounsfield units, HU) of Au@FP nanoparticles at different concentrations (b); confocal images of HUVECs incubated with 200 *μ*g/mL of Au@FP nanoparticles for 2 h the nuclei of cells were stained by DAPI (A), tubulin structure was stained by Alexa Fluor® 488-labeled antibody (B), the images of Au@FP nanoparticles in cells (C), and the merged images (D). Scale bars are 25 *μ*m.

The high atomic number (Z) and X-ray absorption coefficient of the Au elements (5.16 cm^2^/g at 100 keV) [18] enables the Au@FP nanoparticles a good candidate as CT imaging contrast agent. Herein, the CT phantom images and Hounsfield unit (HU) values performance of prepared Au@FP nanoparticles at the concentrations of 5 to 500 *μ*g/mL was studied, and the results are displayed in Figures 2(a) and 2(b). In Figure 2(b), the intensity of CT signal increases rapidly with the sample contents increased. Besides, the CT values have a positive linear relationship with sample concentrations in a dose-dependent manner, and CT signal intensity of Au@FP nanoparticles at 200 *μ*g/mL exhibit much competitiveness with that of a conventional iodine-based contrast agent at the concentration of 300 mg/mL (Figure 2(a)), demonstrating the better enhancement efficacy of Au@FP nanoparticles for CT imaging.

To study the potential of the nanoparticles as fluorescent probes for biomedical NIR imaging, the CLMS was used to image morphology of the HUVECs and track the Au@FP nanoparticles location in cells. As shown in the Figure 2(c), we can see that the cells keep the normal morphology, and the Au@FP nanoparticles were internalised into cells, dispersing uniformly in the cytoplasm and demonstrating bright NIR emission fluorescence imaging, which greatly supports that Au@FP nanoparticles have the potential capability of *in vivo* fluorescence imaging.

In brief, the Au@FP nanoparticles are capable of becoming a CT/Fluorescence bimodality probe for *in vitro* imaging.

### 3.3. Biological Activity of Au@FP Nanoparticles

**Figure 3:**
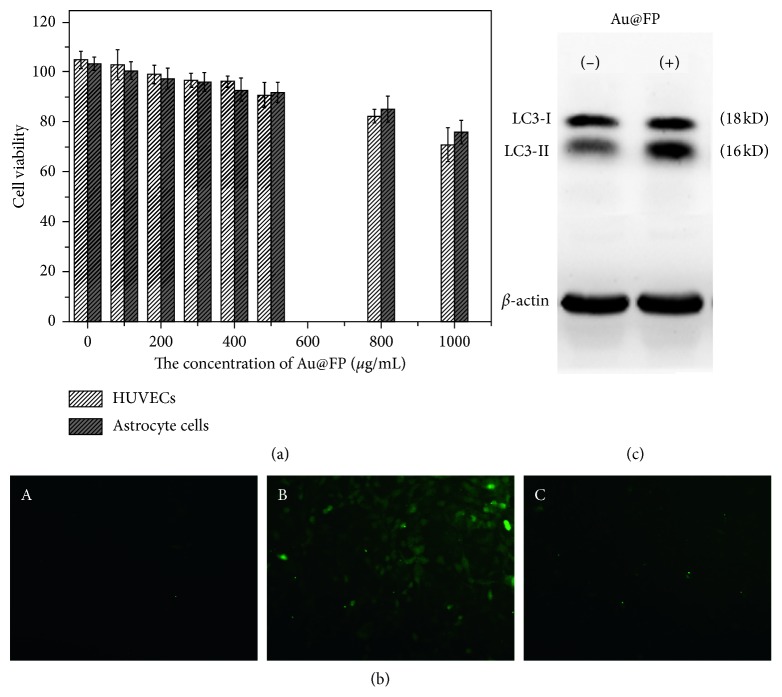
Cell toxicity assay. Measurement of HUVECs and astrocyte cells viability by MTT assay (a); ROS measurement (b): fluorescence images of DCFH in HUVECs control (A), HUVECs incubated with H_2_O_2_ as positive control (B), and treatment with Au@FPs nanoparticles (C). Western blotting with anti-LC3 antibody of HUVECs treatment with Au@FPs nanoparticles and *β*-actin served as the loading control (c).

Safety of nanomaterials should be the first consideration prior to using them in biological applications. Therefore, we investigated the toxicity of as-synthesized Au@FP nanoparticles to astrocyte cells and normal human umbilical vein endothelial cells (HUVEC) by a standard methyl thiazolyl tetrazolium (MTT) assay. From the Figure 3(a), after 24 h of treatment with nanoparticles, we can see that both of cells were not seen to statistically reduce cell viability at the concentration up to 200 *μ*g/mL compared to that of the control, even at the high concentration of 500 *μ*g/mL, and cell viability remained above 90%, demonstrating a high biocompatibility of the Au@FP nanoparticles. Also, the concentration at 200 *μ*g/mL was adopted as the optimum concentration and used in the next work.

To further verify this result, ROS activity assay was carried out to verify cytotoxity induced by the Au@FP nanoparticles. To investigate the ROS activity of Au@FP nanoparticles, the ROS in HUVECs after different treatments were stained with DCFH-DA. For the cells without H_2_O_2_ treatment, almost no fluorescence was observed in the control cells and the cells cultured with Au@FP nanoparticles (200 *μ*g/mL), shown in Figure 3(b). However, the cells pretreated with H_2_O_2_, as the positive control, showed a very strong green fluorescence signal. The intracellular ROS level could be quantified by measuring the fluorescence intensity. Without H_2_O_2_ pretreatment, the ROS in the control cells and the cells cultured with Au@FP nanoparticles (200 *μ*g/mL) were almost at the same level, suggesting that the culture with the Au@FP nanoparticles (200 *μ*g/mL) did not obviously induce ROS generation in normal HUVECs.

It has also been reported that ROS accumulation could cause irreversible cellular damage, thus provoking autophagy in cells, and the autophagy has been considered as emerging toxic mechanism of nanomaterials [35]. Therefore, further investigation was performed to study whether Au@FP nanoparticles are able to induce autophagy in normal HUVECs. It is well known that microtubule-associated protein light chain 3 (LC3) is widely used as an autophagy marker to monitor autophagy [36]. When the autophagy is activated, LC3 will be cleaved at C-terminal to release a cytosolic form LC3-I (18 kD) and then covalently conjugated to the lipid phosphatidylethanolamine (PE) to yield LC3-II (16 kD), an important protein marker for autophagic activity, which further accumulates on the autophagosome membrane [37–39]. In this study, Western blot was performed to detect the special LC3-II protein of HUVECs upon 200 *μ*g/mL of Au@FP nanoparticles treatment compared with the control cells (Figure 3(c)). Collectively, these results strongly supported that Au@FP nanoparticles could induce the upregulation of LC3-II, indicating that Au@FP nanoparticles were able to induce autophagy in HUVECs. In consideration of results of MTT and ROS activity assay that the 200 *μ*g/mL of Au@FP nanoparticles exhibit the negligible cytotoxicity compared with control cell, the Au@FP nanoparticle-induced autophagy promotes cell survival and acts as a positive cellular survival mechanism in the normal HUVECs [40].

On the basis of above, our prepared Au@FP nanoparticles have satisfactory biocompatibility and hold great potential for biomedical applications.

### 3.4. *In Vivo* CT Imaging and NIR Fluorescence Imaging

**Figure 4:**
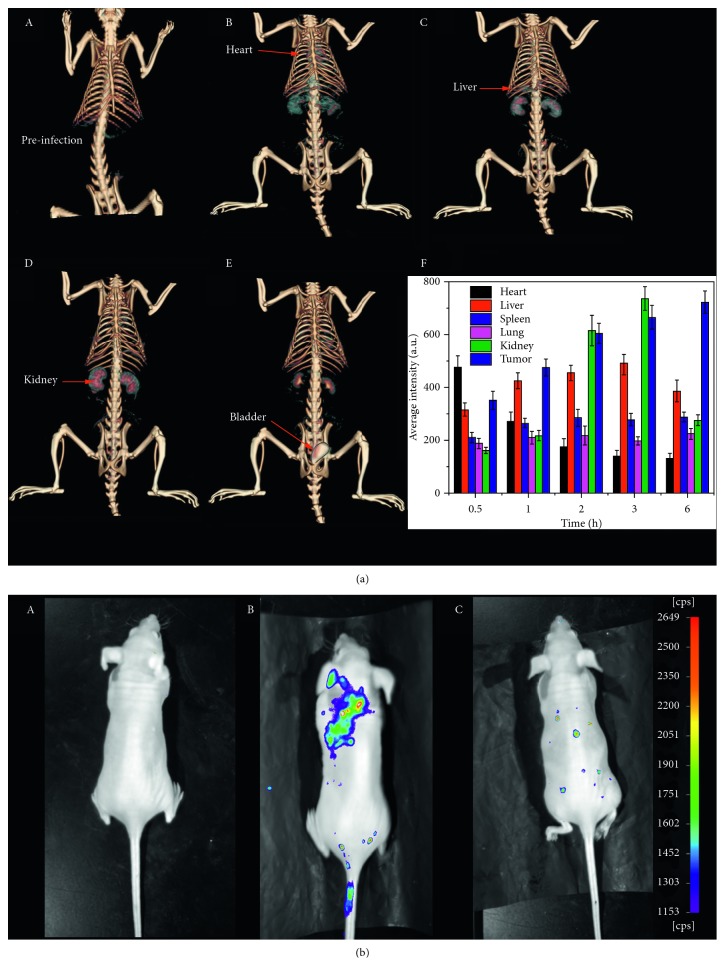
*In vivo* CT images of SD rat before injection (a) and after injection with Au@FP nanoparticles via the tail vein at different times: before injection (A), and one min (B), 0.5 h (C), 3 h (D), 6 h (E) after injection and the HU average intensity of major organs (heart, liver, spleen, and lung kidney) after intravenous injection of Au@FP nanoparticles at different timed intervals (F); (b). NIR fluorescence light image of SD rat before injection (A) and 3 h (B) and 6 h (C) after injection.

For *in vivo* CT imaging, CT imaging of SD rats was recorded at different time points after Au@FP nanoparticles injection. Compared with the image before injecting, a great contrast enhancement was observed in the mouse body. At timed intervals, the evident enhancement of the signals in different organs could be seen in Figure 4(a). The contrast agent first appeared in the heart in the first min after injection (Figure 4(a)B). It is very fast and difficult to be recorded, which showed that the Au@FP nanoparticles can enhance CT imaging in the circulating system, running smoothly in the blood without any aggregation [41]. Then, from the 3D-renderings of CT images shown in the Figure 4(a)C, we can see that the organs of live and the renal cortex were lighted by Au@FP nanoparticles at 0.5 h, the CT signals of kidney imaging were greatly enhanced 3 h after injection, shown in the Figure 4(a)D, and the CT contrast intensity in the body and organs obviously decrease, while CT images of the bladder became clear (Figure 4(a)E), showing the excellent renal clearance properties of Au@FP nanoparticles, which is attributed to their optimal particle size and surface functionalization. As the consistent evidence, the evident CT signals in different organs were also recorded and are shown in Figure 4(a)F, at timed intervals, and HU value of kidney rose from 167 to 736 and remained at 276 at 6 h after injection, while the signal value of heat showed the obvious decrease over time from 576 to 142. The signal value in other organs shows little fluctuation, which are all consistent with the CT imaging. In summary, all of the results demonstrate that the synthesized Au@FP nanoparticles as CT contrast agent candidate have longer blood circulation time, good biocompatibility, and better CT imaging ability.

The *in vivo* NIR fluorescent images were also collected before injection and 3 h and 6 h after injection (Figure 4(b)). Various colors including red, orange, yellow, green, and blue correspond to the successive decrease in fluorescence intensity. At first, NIR fluorescence imaging showed that the Au@FP nanoparticles were dispersed over the whole body at 3 h after injection (Figure 4(b)B), exhibiting the Au@FP nanoparticles could be rapidly dispersed in the blood without any aggregation and running in the circulating system. As time elapses, the fluorescence intensity in the majority of organs greatly decreases. While comparing the preinjection images with those collected 6 h after injection (Figure 4(b)C), we could not see any obvious difference, besides the autofluorescence of rats. The bright NIR fluorescent imaging and long circulation time and good clearance properties showed the excellent NIR fluorescent imaging ability of Au@FP nanoparticles, and it can provide the real-time and high-sensitivity imaging for future clinical surgical applications.

### 3.5. *In Vivo* Toxicity

**Figure 5:**
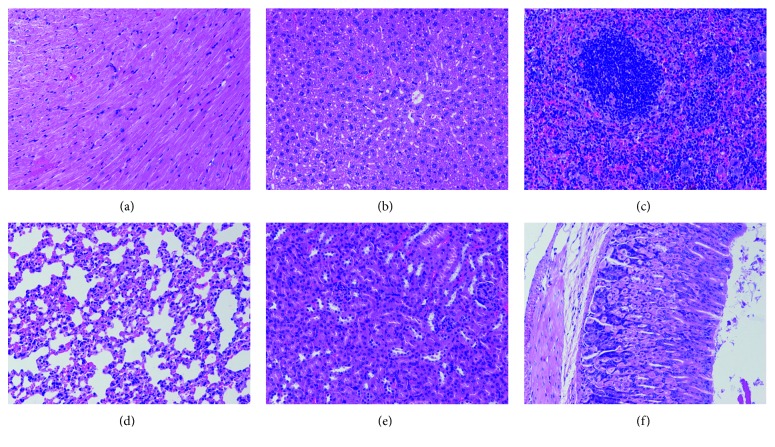
H&E staining of the organs sections ((a) heart; (b) liver; (c) spleen; (d) lung; (e) kidney; (f) stomach) harvested from mice after 14 d intravenous injection of Au@FP nanoparticles.

In order to further investigate the potential *in viv*o toxicity of Au@FP nanoparticles on the treated mice, we carried out the histochemical analysis and biochemical analyses of blood. As shown in Figure 5, the mice show negligible tissue damage of major organs (heart, liver, spleen, lung, kidney, and stomach) after 14 d intravenous injection of Au@FP nanoparticles with the dose of 200 *μ*L (200 *μ*g/mL). The serum biochemistry assay of Au@FP nanoparticles was also performed by renal functional test, and the results are shown in Table 1. Compared with control groups, as renal functional parameters, the value of creatinine (Cr), blood urea nitrogen (BUN), and uric acid (UA) do not reveal any dysfunction in terms of renal function. Overall, the preliminary *in vivo* toxicity results suggest that Au@FP nanoparticles present great promise as a nontoxic nanoprobe in the field of bimodality bioimaging.

## 4. Conclusions

In summary, we have successfully developed a unique and promising CT/fluorescence Au@FP nanoprobe for *in vitro* and *in vivo* imaging. The nanoprobe was facile to be fabricated via one-step solvent-mediated self-assembly method based on Au nanoparticles and NIR emissive semiconducting fluorescence polymers. Owing to their nontoxic composition, smart sizes, and surface modification, *in vivo* results demonstrate that the Au@FP nanoparticles have good cytocompatibility and enhancement of the autophagy inducement, long blood circulation, unconspicuous *in vivo* toxicity, and excellent fluorescence and CT imaging effects. To our knowledge, it is rarely reported that Au nanoparticles and NIR emissive semiconducting fluorescence polymers were assembled together to provide simultaneous CT imaging and NIR fluorescence imaging. Overall, the studies assuredly indicate the significant potential application of Au@FP nanoparticels as a bimodality noninvasive fluorescence/X-ray CT contrast agent for *in vivo* biomedical imaging and diagnosis.

## Figures and Tables

**Table 1:** Serum biochemistry assay for renal function on the levels of BUN, Cr, and UA before injection and at 24 h after injection of Au@FP nanoparticles. (*n* = 5).

	Before injection	After injection
BUN (mmol/L)	10.12 ± 0.35	9.98 ± 0.12
Cr (*μ*mol/L)	30.11 ± 0.34	29.67 ± 0.52
UA (*μ*mol/L)	76.06 ± 0.25	73.00 ± 0.85

## Data Availability

All data generated or analyzed during this study are included in this published article.
